# Experiences when implementing enhanced cognitive behavioral therapy as a standard treatment for anorexia nervosa in outpatients at a public specialized eating-disorder treatment unit

**DOI:** 10.1186/s40337-022-00536-7

**Published:** 2022-02-05

**Authors:** Ute Kessler, Malin Mandelid Kleppe, Guro Årdal Rekkedal, Øyvind Rø, Yngvild Danielsen

**Affiliations:** 1 Division of Psychiatry, Haukeland University Hospital, Bergen, Norway; 2 Department of Clinical Medicine, University of Bergen, Bergen, Norway; 3 Department of Clinical Psychology, University of Bergen, Bergen, Norway; 4 Regional Department for Eating Disorders, Division of Mental Health and Addiction, Oslo University Hospital, Oslo, Norway; 5 Institute of Clinical Medicine, Faculty of Medicine, University of Oslo, Oslo, Norway

**Keywords:** Anorexia nervosa, Enhanced cognitive behavioral therapy, CBT-E, Treatment dropout

## Abstract

**Background:**

Enhanced cognitive behavioral therapy (CBT-E) is a promising treatment option for outpatients with anorexia nervosa (AN). We aimed to determine the effectiveness of CBT-E as a standard treatment for adult outpatients with AN from the specialized eating-disorder unit of a public hospital with responsibilities to their catchment area.

**Methods:**

This study had an open, longitudinal design. Thirty three (of planned 100) outpatients aged > 16 years suffering from AN were included to receive 40 sessions of CBT-E. Eating-disorder psychopathology and body mass index (BMI) were assessed before and after treatment, while comorbid psychiatric symptoms and trauma experiences were evaluated at the baseline, and therapeutic alliance was assessed after 4 weeks of treatment.

**Results:**

A high proportion (69%) of patients dropped out of the treatment. Patient recovery was considered when they reached BMI > 18.5 and Eating Disorder Examination Questionnaire (EDE-Q) score < 2.5, and 27% of all patients recovered.

**Conclusions:**

Patients who completed the treatment had mostly satisfactory outcomes. Considering the high dropout rate, it is necessary to improve the strategies for engaging patients in therapy. Several aspects of CBT-E as a standard treatment are discussed regarding the high dropout rate.

*Trial registration* ClinicalTrials.gov. Identifier: NCT02745067. Registered: April 20, 2016. https://clinicaltrials.gov/ct2/showNCT02745067

## Plain English summary

Anorexia nervosa (AN) is difficult to treat, and no specific treatment approach has been demonstrated to be superior for adult outpatients. However, outcome data indicate that enhanced cognitive behavioral therapy (CBT-E) is a viable and promising treatment option for adults with AN, and its efficacy has been indicated in cohort studies and randomized controlled trials. The present study aimed to determine the effectiveness of CBT-E as a standard treatment for adult outpatients with AN from the specialized eating-disorder unit of a public hospital with responsibilities to their catchment area. Outpatient CBT-E was administered to 33 patients in a course of 40 sessions. Although 27% of the included patients had satisfactory treatment outcomes (healthy weights and reduced eating-disorder symptoms), more than two-thirds of all patients terminated treatment early. Several aspects of CBT-E as a standard treatment are discussed regarding this high dropout rate.

## Introduction

No specific treatment approach has been demonstrated to be superior for adult outpatients with anorexia nervosa (AN) [1–3]. However, outcome data indicate that enhanced cognitive behavioral therapy (CBT-E) is a viable and promising treatment option for adults with AN [4], and its efficacy has been demonstrated in cohort studies [5] and randomized controlled trials [6, 7]. In the SWAN (Strong Without Anorexia Nervosa) study, 120 patients with AN were randomized to receive Specialist Supportive Clinical Management (SSCM), Maudsley Model of Anorexia Nervosa Treatment for Adults (MANTRA), or CBT-E [7]. Treatments were completed by 60% of patients. The three approaches produced equivalent effects on eating-disorder psychopathology; however, CBT-E was superior in helping patients to reach a healthy weight by the 12-month follow-up. The NICE (National Institute for Health and Care Excellence) guideline for eating disorders recommends MANTRA, eating-disorder-focused cognitive behavioral therapy (CBT), or SSCM for adults with AN. Further, eating-disorder-focused CBT is the recommended treatment for non-underweight eating disorder patients [8]. These guidelines and studies suggest that CBT-E could be a useful approach for treating AN in adult outpatients.

As a specialized treatment center for eating disorders, we are aiming to provide evidence based treatment. The promising results of the aforementioned studies supported the implementation of CBT-E as a standard treatment for all adult outpatients starting AN treatment at our center, with few exceptions. Learning one treatment approach effectively with supervision from experienced CBT-E therapists was considered essential for successful implementation. In sum, CBT-E was established as the only approach recommended for most of the referred patients.

A crucial question is whether results from research settings can be transferred into real-world situations [9]. Challenges in everyday practice might include balancing a flexible enough adjustment to patients’ preferences and needs, and at the same time preventing drift from core aspects of the treatment protocol [10, 11]. Previous studies have often been carried out in research departments with more resources and without responsibilities to their catchment areas, which might influence patient selection. In real world settings, challenges may for instance occur when patients deviate from the majority of participants in clinical trials with regard to e.g. culture, etiology of the eating disorder or symptom presentation [12]. Therefore it is also important to replicate study results in different cultural settings and by clinicians not associated with those who developed the treatment model. Previously, we performed a pilot study to assess the effectiveness of CBT-E in our public outpatient eating-disorder unit with responsibilities to the catchment area [13]. Although half of the 44 included patients did not complete CBT-E, those who did, achieved a significantly large BMI increase at 1 year after the start of therapy (mean BMI change 2.9 ± 2.3 kg/m^2^). However, eating-disorder symptomatology and other factors potentially impacting treatment outcomes were not assessed [13]. The primary aim of the present study was therefore to determine the effectiveness of CBT-E as a standard treatment for AN in adult outpatients, as well as therapeutic alliance and baseline predictors of treatment dropout and other outcomes [14]. Our secondary aim was to evaluate our experiences with the aforementioned implementation of CBT-E.

## Methods

### Setting

In Norway, the government has the responsibility for offering health care to the whole population, regardless of socioeconomic status or place of living. There is a tax funded universal health coverage (no fee for inpatient treatment, and all costs for outpatient treatment exceeding in total 2460 NOK (about 275 $) within a year are covered). Further, psychiatric services are public and available to everyone, and patients in the catchment area are admitted from general practitioner (GP) to the local secondary health care center (District psychiatric center). District psychiatric centers provide less specialized services on a decentralized level. Tertiary health care is provided at highly specialized hospital units, where patients are referred to from the District psychiatric center (secondary care). The present study had an open, longitudinal design, and included patients from the Department of Eating Disorders at Haukeland University Hospital, Bergen, Norway. This is a standard clinical unit at a tertiary-level eating-disorder center with responsibilities to the catchment area. The clinic has an interdisciplinary team that specializes in eating disorders for adolescents and adults. The clinic is considered responsible for the most severely ill patients with eating disorders in its region. This includes patients that have previously undergone, but not successfully responded to, eating-disorder treatments in specialized secondary or tertiary health care. The clinic also runs training programs for CBT-E at the secondary health care level. Most patients are referred to the clinic from mental health practitioners from secondary health care centers.

This study employed nine CBT-E therapists from our unit, all of which were clinical psychologists. To implement CBT-E, substantial efforts were made to train staff in the delivery of CBT-E, and all team members attended a 2-day CBT-E workshop performed by the treatment developer Christopher Fairburn, followed by regular supervision by him. New team members also received weekly individual supervision from an experienced on-site CBT-E therapist during their first year at the clinic. Implementing CBT-E for individual patients was discussed in 2-h-long weekly team meetings. One of the main focuses of these meetings was ensuring that all therapists were following the CBT-E manual [15].

All outpatients received about 40 sessions of CBT-E that covered the different treatment sessions for underweight patients described in the CBT-E manual (broad version if indicated) [15], which was the standard AN treatment at our clinic. However, during our study period, hospital guidelines changed, demanding a wider repertoire of treatments offered. The impact of this decision on our treatment trial is elaborated on in the discussion.

### Participants

Treatment was offered to patients who either had received at least one unsuccessful treatment attempt in a secondary health care unit or had severe AN evaluated as not manageable in a secondary health care service unit. All patients who consented to receive CBT-E for AN between December 2016 and 2019 were asked to participate in the study. We aimed to study 100 patients chosen by applying the following inclusion criteria [14]: those aged > 16 years who had AN diagnosed by a clinical assessment based on the DSM-5 (Diagnostic and Statistical Manual of Mental Disorders, Fifth Edition) performed by an experienced clinical psychologist [16]. Patients were excluded if outpatient treatment was deemed unsafe or if they had psychiatric comorbidities that preclude a focused eating-disorder treatment, such as psychosis or drug abuse. There was no lower limit for BMI as long as the medical doctor and clinical psychologist considered outpatient treatment to be safe.

### Assessment

Patients were assessed at the baseline (before starting treatment) and at the end of treatment (completed CBT-E or dropped out). The following self-report questionnaires were used: eating-disorder psychopathology was assessed using the Eating Disorder Examination Questionnaire (EDE-Q) [17], and the severity of psychosocial impairment associated with the eating disorder was measured using the Clinical Impairment Assessment Questionnaire (CIA) [18]. The Global Severity Index (GSI) from the Symptom Checklist-90–Revised (SCL-90) was used to evaluate overall psychological distress [19], Beck Depression Inventory-II and the Beck Anxiety Inventory were used to assess the severity of depressive and anxiety symptoms [20, 21]. Trauma experiences were self-evaluated at baseline by patients answering questions about exposure to physical, emotional, and sexual abuse. The relationship between patient and therapist and their agreement on tasks and goals for the therapy were assessed at 4 weeks into the treatment using the Working Alliance Inventory–Short Form (WAI-SF) [22], which is a 12-item self-reporting measure with different versions for the patient and therapist.

The assessment also included demographic variables, self-reported age when eating-disorder symptoms began, previous treatment attempts, comorbid psychiatric diagnoses as assessed by the Mini-International Neuropsychiatric Interview (MINI) [23], self-reported self-harm during the year prior to inclusion, and therapist-reported reasons for dropout.

Weight was measured before treatment and at the end of treatment (dropout or completed CBT-E) using a beam-balance scale. Height was measured using a wall-mounted height board.

### Statistics

Data analyses were performed using SPSS (version 26). The characteristics of those patients who completed or dropped out of treatment were compared using non-parametric Mann–Whitney U tests and Fisher’s exact chi-square tests for continuous and categorical variables, respectively. The phi coefficient was used to quantify the effect sizes of group differences in categorical variables and *r* in continuous variables, for which values of 0.1, 0.3, and 0.5 are considered small, moderate, and large effects, respectively [24]. Due to small sample size, Wilcoxon signed-ranks tests were used for the longitudinal analyses of BMI changes and eating-disorder symptoms.

### Ethics

This study was approved by the Regional Committee for Medical and Health Research Ethics, Western Norway (REK Vest 2015/2328). All patients provided written informed consent prior to participation.

## Results

**Fig. 1:**
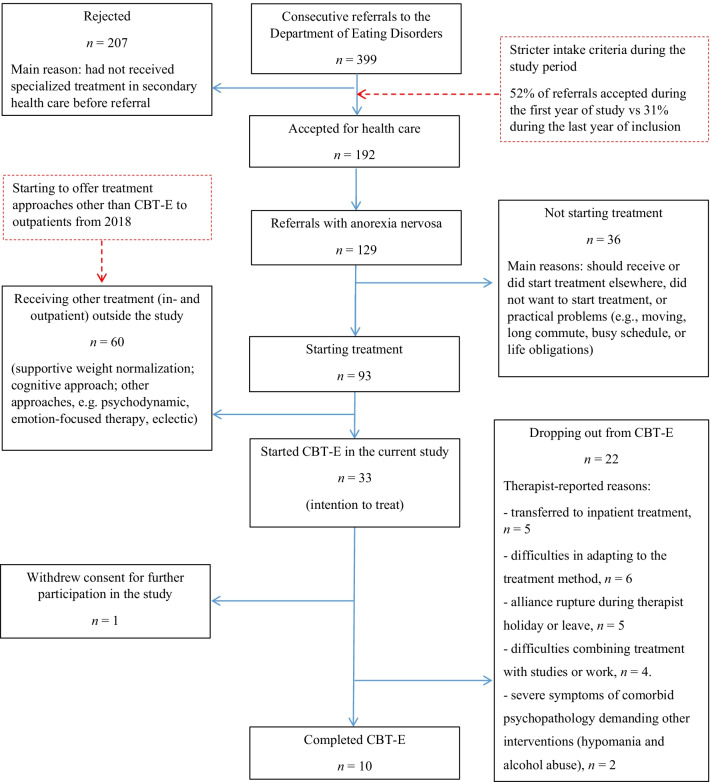
Study flow chart

Of the 399 patients referred to our clinic from December 2016 to 2019, 192 (48%) were accepted. Reasons for denying a referral were most commonly secondary health care treatment for eating disorders having not yet been attempted, or that treatment should have been offered in secondary health care. The percentages of accepted referrals were 52% and 31% during the first and last years of inclusion, respectively. Among the accepted referrals, 129 were diagnosed with AN. Of these, 93 patients agreed to start the treatment after an initial assessment phase. Reasons for not starting treatment were most commonly that the clinical psychologist considered that treatment should be provided elsewhere, patients declining the offer, and practical problems or other patient obligations that made prioritizing treatment difficult. 60 started treatment as in- or outpatients at our department outside the current study. This included supportive weight normalization, cognitive approaches and other treatment approaches, e.g. psychodynamic, eclectic or emotion-focused therapy. Figure 1 shows the study flow chart. Only 4 patients from 2019 could be included, and the study ceased after 3 years, including a total of 33 patients (out of the 100 planned).

**Fig. 2:**
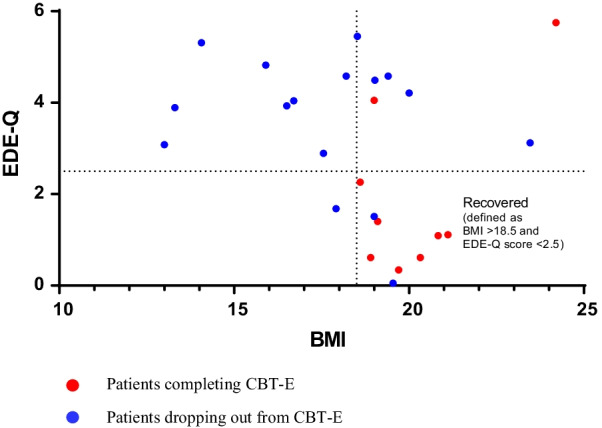
Post-treatment eating disorder symptoms (EDE-Q score) and BMI for patients completing and dropping out from CBT-E. Data from 25 of 33 patients who provided BMI and EDE-Q data at the end of treatment or dropout. EDE-Q, Eating Disorder Examination Questionnaire; BMI, body mass index; CBT-E, enhanced cognitive behavioral therapy

Table 1 lists the baseline demographics, clinical variables, and comparisons between patients who completed treatment and those who left treatment prematurely. One of the included patients withdrew their consent during the study, on which no additional information was available. Among the remaining 32 patients, 10 completed treatment, and 9 provided CIA and EDE-Q data (Table 1). A high proportion (69%, *n* = 22) dropped out of treatment. Eight patients (24%) ceased treatment early (fewer than 10 CBT-E sessions), and another 24% after fewer than 20 sessions. Of the 22 patients who dropped out, 16 provided EDE-Q and BMI data. Figure 2 illustrates the EDE-Q and BMI data at the end of treatment or time of dropout. Of the 25 patients who provided posttreatment data, 9 (36%) recovered, defined as BMI > 18.5 and EDE-Q score < 2.5 (cutoff based on Norwegian data [25]). Two of these patients terminated their treatment prematurely, and the remaining seven completed the treatment. Comparing BMIs at the baseline and at the end of treatment or dropout using Wilcoxon signed-ranks tests revealed that BMI increased significantly in both groups during treatment (completed: *z* =  − 2701, *p* = 0.007, *r* = 0.85; dropout: *z* =  − 1,964,531, *p* = 0.049, *r* = 0.42), whereas a reduction in eating-disorder symptoms was observed in the completer group only (completed: *z* =  − 2.310, *p* = 0.021, *r* = 0.77; dropout *z* =  − 1.603, *p* = 0.109, *r* = 0.40). Further, those who dropped out had significantly worse outcomes, as measured by both the percentage of patients who recovered (12.5% vs 78%) and by comparing changes in BMI or EDE-Q score (Table 1). Of all the included patients, 27% recovered (i.e., BMI > 18.5 and EDE-Q score < 2.5).

**Table 1:** Clinical characteristics of 33 patients with AN and comparisons between patients who completed CBT-E and those who left treatment prematurely

	Whole group *n* = 33			Patients who completed *n* = 10			Patients who dropped out *n* = 22			Group comparison		
	Median	Range	IQR	Median	Range	IQR	Median	Range	IQR	U	*p*	*r*
BMI pre	16.5	12.5–21.5	3.4	17.2	13.8–21.5	2.5	16.1	12.5–19.3	3.2	81.5	0.251	0.20
Pre-post change in BMI	0.9^a)^	− 2–10.4	2.7	2.7	− 0.4–10.4	3.1	0.2	− 2.0–5.7	1.9	**52.0**	**0.018**	**0.42**
EDE-Q global pre	4.0	0.8–5.7	2.1	3.4	1.8–5.5	2.2	4.2	0.8–5.7	1.7	83.0	0.287	0.19
Pre-post change in EDE-Q	0.6^b)^	− 4.3–1.2	2.1	2.4^c)^	− 4.3–1.0	2.7	0.4^d)^	− 2.2–1.2	0.9	**31.0**	**0.020**	**0.46**
CIA total pre	37	13–48	16	35	17–48	26	39	13–48	13	92.5	0.483	0.13
Pre-post change in CIA	**− **8^b)^	− 30–22	15.5	**− **16^c)^	− 30–− 1	19	**− **5^d)^	− 24–22	13.8	**24.0**	**0.005**	**0.54**
Age	20.0	16–49	8	19	16–49	11	22.5	17–38	8	92.0	0.483	0.13
Duration of illness	7.0	1–34	7	7.5	1–33	6.5	7.0	1–34	7.3	108.5	0.952	0.01
BAI pre	16.5^a)^	3–62	19	11	3–62	21	21^e)^	5–43	19	86.5	0.441	0.14
BDI pre	31	2–60	22	25	2–57	22	35	15–60	19	78.0	0.204	0.23
SCL-90 GSI pre	1.3	0.3–3.5	1.4	1.2	0.3–3.5	1.5	1.6	0.4–2.7	1.5	88.5	0.388	0.15
WAI-SF patient	70^f)^	43–84	10	77^c)^	59–84	15	69^d)^	43–76	14	**36.0**	**0.043**	**0.41**
WAI-SF therapist	72^b)^	41–81	10	78^c)^	68–81	8	70^g)^	41–77	14	**19.5**	**0.003**	**0.59**
Number of CBT-E sessions	20.5^a)^	2–78	32	40.5	26–78	30	12.5	2–57	20	**21.5**	** < 0.001**	**0.64**

	*n*	%	*n*	%	*n*	%	Chi-Square	*p*	Phi
Duration of illness > 6 years	20	60.6	7	70.0	12	54.4	0.681	0.467	0.146
Severe AN (BMI < 16)	12	36.4	2	20.0	10	45.5	1.901	0.248	0.244
PTSD	6	18.2	0	0.0	6	27.3	3.357	0.142	0.324
Depression, previous	24	72.7	6	60.0	17	77.3	0.390	0.606	0.118
Depression, current	15	45.5	4	40.0	11	50.0	0.276	0.712	0.093
Current antidepressant use	14	42.4	1	10.0	12	54.5	**5.656**	**0.024**	**0.420**
Physical abuse	5	15.2	1	10.0	4	18.2	0.349	0.656	0.104
Emotional abuse	9	27.3	2	20.0	7	31.8	0.475	0.681	0.122
Sexual abuse	7	21.2	1	10.0	6	27.3	1.200	0.387	0.194
One or more traumas	14	42.4	3	30.0	11	50.0	1.117	0.446	0.187
Self-harm (during the previous year)	13	39.4	1	10.0	11	50.0	4.693	0.050	0.383
Previous outpatient treatment at secondary treatment unit for eating disorders	24	72.7	8	80.0	15	68.2	0.475	0.681	0.122
Previous treatment at specialized eating-disorder unit	8	24.2	2	20.0	6	27.3	0.194	1.000	0.078
Previous admission to somatic or psychiatric hospital for eating disorders	15	45.5	3	30.0	11	50.0	1.117	0.446	0.187

^a)^ *n* = 32, ^b)^ *n* = 25, ^c)^ *n* = 9, ^d)^ *n* = 16, ^e)^ *n* = 21, ^f)^ *n* = 26, ^g)^ *n* = 15

*p* from independent-samples *t*-tests and Fischer’s exact tests, boldface: *p* < 0.05

AN, anorexia nervosa; CBT-E, enhanced cognitive behavioral therapy; BMI, body mass index; IQR, Interquartile range; EDE-Q, Eating Disorder Examination Questionnaire; CIA, Clinical Impairment Assessment Questionnaire; BDI, Beck Depression Inventory-II; BAI, Beck Anxiety Inventory; SCL-90 GSI, Global Severity Index from the Symptom Checklist-90–Revised; PTSD, posttraumatic stress disorder; WAI-SF, Working Alliance Inventory–Short Form

After 4 weeks of treatment, those who completed treatment reported significantly higher WAI-SF scores compared with those who ceased treatment prematurely, with a large effect size (Table 1). Table 1 lists data on depression and anxiety symptoms. Patients who ceased treatment prematurely had a greater likelihood of using antidepressant medications.

Therapist-reported reasons for dropping out are as follows: Five patients (23% of all dropouts) were transferred to in-patient treatment due to their increased need for support to gain weight. Six patients (27%) were reported to have dropped out early due to difficulties in adapting to the treatment method, and five (23%) dropped out after alliance rupture due to the therapist leaving for either a vacation or a change of workplace. Four patients dropped out due to difficulty in combining treatment with studies or work, and two patients dropped out due to comorbid illness (hypomania and alcohol abuse).

After offering treatment approaches other than CBT-E to outpatients in our clinic from 2018, the percentage of patients ceasing treatment prematurely did not improve (14/22 patients included in 2016/2017 dropped out compared with 8/10 included in 2018/2019) (*p* > 0.05).

## Discussion

The present study originally aimed to determine the effectiveness of CBT-E in patients with AN referred to ordinary clinical treatments at a specialized eating-disorder unit with responsibilities to its catchment area, as well as the baseline predictors of treatment outcomes and dropout. The three main results obtained in this study are as follows: Firstly, a high dropout rate was observed, with 22 of 32 patients prematurely ceasing treatment. These patients had a greater likelihood of using antidepressant medications, but no significant differences were found for other psychiatric comorbidities. Secondly, those who dropped out had more unfavorable treatment outcomes with lower BMIs and higher EDE-Q scores than those who completed treatment. This highlights the need to prevent patients from ceasing treatment early. Only nine patients recovered, demonstrating that most of the included patients did not receive sufficient help in managing their eating disorder. However, those who completed treatment had mostly satisfactory treatment outcomes. Thirdly, we included fewer than the intended number of patients and were therefore unable to answer the scientific questions regarding baseline predictors for treatment outcomes and dropout.

Studying AN treatment is challenging, with its complex comorbidity and medical instability hindering the inclusion of patients in studies [1]. Our difficulty in recruiting a sufficient number of patients was therefore not surprising. The small number of study participants prevented us from answering our research questions; however, our secondary aim was to evaluate our experiences when implementing CBT-E, which was the only recommended treatment option at our clinic during the first part of the study.

The competency in delivering CBT-E and treatment fidelity were enhanced through workshops with the developer of the manual, weekly supervision, and weekly staff meetings to discuss implementation. Despite this effort, a large percentage of patients prematurely ceased treatment before they fully recovered. Premature dropout is a common problem in treating eating disorders [26]. In our previous implementation study performed in 2013 and 2014, 50% of patients did not complete treatment [13]. In the present study, 69% ceased treatment prematurely (53% after excluding patients referred to inpatient treatment from the dropout group), compared with 36% in the UK-Italy study [5], and 22% in the Anorexia Nervosa Treatment of OutPatients (ANTOP) study [6]. In a study performed in Western Australia, which was the first published CBT-E trial that included patients with a BMI < 17.5, 12 of 34 (35%) patients with AN completed the treatment [27], whereas a recent study involving the National Health Service in London reported that 54% of patients with AN completed CBT [28]. Results from different studies and settings are not directly comparable due to differences in patient samples and definitions of dropout [29]; for example, transfer to inpatient treatment is only included in some studies. In the ANTOP study, 35% of patients in the CBT-E group received residential crisis interventions during the trial and were not categorized as dropouts [6]. In our study, patients receiving intermittent in-patient care were categorized as dropouts from CBT-E. Further, most patients referred to our clinic had already undergone at least one unsuccessful treatment attempt at a secondary health care unit before being referred to our clinic. Such differences should be considered when comparing results between similar studies. Compared to previously published RCT’s, our study included patients with BMI below the lower limit (BMI 14 and 15) in the ANTOP study and Byrne et al., respectively [6, 30]. Beyond this, there are small differences in the exclusion criteria and the clinical characteristics regarding comorbidity. Our impression, however, is that the recruiting procedure and patient selection might also impact the treatment results. For example, patients in the present study stemmed from consecutive referrals to a tertiary treatment unit after treatment failure in secondary health care, whereas patients in the ANTOP study were recruited also via advertising in local and regional media before assessment and further referral to the tertiary unit [31], illustrating that the recruiting procedure might contribute to different selection of patients in studies. Due to the special conditions of the Norwegian health care system, our patients are selected in terms of treatment resistance and severity of illness, but not socioeconomic status. Roughly seen, the results of the current study are in line with the findings presented in a review by Waller on replication of efficacy results from RCTs in everyday practice (effectiveness) [10], stating that the clinical outcomes are very close to those found in research trials, but with higher attrition rates. The effect of cognitive therapy is weaker in patients with AN (only approximately 30% recovery rate) compared to non-underweight eating disorder patients [10], in line with the findings from our study (28% recovered).

Despite the satisfactory results regarding patients adhering to full treatment, the high dropout rate was disappointing and appears to be the main obstacle when delivering CBT-E in this setting. We have therefore reflected upon why so few of the patients accepted for CBT-E had successful treatment, by considering questions such as are there structural reasons for our difficulties in offering effective treatment, and can patient-, treatment-, or therapist-related factors explain the high dropout rate?

### Structural and therapist-related reasons

In the present study, possible structural reasons for the high dropout rate included the changes in our clinic that impacted the treatment setting. During CBT-E implementation, we initially accepted patients with few comorbid symptoms for training purposes. Stricter intake criteria were applied from 2018, resulting in a lower percentage of accepted referrals at our tertiary clinic and more eating-disorder patients with less-complex comorbidities receiving treatment in secondary health care. The number of patients accepted at our clinic during recent years has therefore reduced, and they mostly consisted of patients with complex comorbidities, increasing the burden of eating disorders. The patients in this study were characterized as being more complex compared with previous years by the clinical psychologists, due to increased presence of maladaptive personality traits, interpersonal difficulties, and trauma-related symptoms.

Alongside the stricter intake criteria, and in order to disseminate CBT-E into first- and second-line medical care in Western Norway, more than 100 psychotherapists in our catchment area received CBT-E training from experienced CBT-E therapists in our clinic during this period. As a consequence, most of the treatment units that referred patients to our tertiary care unit already had structured educational groups supervised by our trained clinicians, with the goal of teaching and establishing CBT-E. We therefore experienced changes in our clinical setting, with a greater focus on treating patients who had already received unsuccessful AN treatment in secondary health care.

At the same time, our clinic was recommended to offer a wider spectrum of treatment protocols. While initially being the only outpatient treatment option available, CBT-E was now offered after evaluating the complexity of the eating disorder, earlier treatment attempts, and the experiences of the patients with CBT-E. Other treatments than CBT-E were considered when the patient already had several failed CBT-E attempts, or the patient had a strong preference for other treatments. However, the stronger focus on selecting patients for CBT-E did not improve the dropout rate among the included patients.

Focusing solely on CBT-E helped to establish the method and properly provide an effective specific treatment approach focusing on weight gain as well as thoughts and behaviors maintaining AN. It also prevented the clinicians from being unsystematic and unfocused. However, a tertiary treatment unit with responsibilities to its catchment area and disseminating CBT-E to second-line treatment units should be organized to allow for several evidence-based treatments.

A high level of expertise must be maintained over time for successful eating-disorder treatment. During the present study we observed a high turnover of therapists at our unit, making the therapeutic environment vulnerable due to the difficulty in replacing these experts. In fact, 23% of dropouts were reported as being caused by a change to the assigned therapist during a holiday or other change of staff. This occurred despite applying the “system of understudying” [15] and a deliberate strategy of not starting treatment closer than 8 weeks before the start of a summer holiday, Christmas vacation or therapist leave, and careful planning for the co-therapist before vacations (e.g., using the same scales for monitoring weight). Further, weaker therapeutic alliances were related to premature dropout in our study. Together these factors highlight the importance of a stable therapeutic alliance and providing frequent AN treatment sessions. However, as highlighted in a previous review, the therapeutic alliance during eating-disorder treatment should not compromise the adherence to treatment components necessary for recovery (e.g., focusing on weight monitoring and weight gain), since early symptom improvements are important for the subsequent alliance [10].

### Patient-related reasons

Most patients in the current study had failed to respond to previous treatments, about one third had severe AN (BMI < 16) and almost to thirds enduring AN (duration of illness > 6 years). More than 40% of the included patients reported being exposed to previous traumatic events or experiences, and 18% had been diagnosed with posttraumatic stress disorder (PTSD). Subsequent psychological reactions to traumatic experiences and interpersonal difficulties represent significant obstacles to successful eating-disorder treatments, and are associated with high dropout rates and prolonged illness [32]. CBT-E lacks an integrated approach for stabilizing trauma. We therefore initially hypothesized that the high dropout rate was attributable to the inability to handle trauma-related symptoms arising during eating-disorder treatment; unfortunately, there was insufficient data to further elaborate on this. Although CBT-E has shortcomings in handling trauma-related symptoms, therapist-reported dropout reasons indicated that this was not the crucial factor for patients ceasing treatment prematurely. We suggest that most dropouts were not related to the treatment method, instead being due to the severity and complexity of the illness and the ambivalence toward treatment, which is inherent in AN. However, the clinicians experienced that the treatment approach was too narrow for some patients and did not address their specific needs, especially for those struggling with severe consequences of traumatic experiences. Of the 7 patients reporting sexual abuse and 14 reporting traumatic experiences, 6 and 11 patients ceased treatment prematurely, respectively. Although this difference was not statistically significant, it may indicate that the treatment was particularly challenging for this patient subgroup. We therefore highlight the need for establishing more-specific guidance on applying CBT-E to patients struggling with trauma reactions during AN-treatment. Mitchell and colleagues [33] acknowledged the bidirectional relationship between trauma/PTSD and eating disorders, and developed an integrated type of cognitive behavioral theory that may account for the persistence of comorbid PTSD and eating disorders. Addressing trauma-related symptoms might increase the proportion of patients who respond well to AN treatment [34, 35].

Our experience was that patients referred to a tertiary treatment unit with responsibilities to its catchment area due to therapy failure had a high comorbidity burden. Research guiding the treatment of eating disorders with complicated psychiatric comorbidities is currently sparse. In order to improve treatment retention and outcomes, using more-adaptive treatment strategies for subgroups of patients with AN in future studies should include assessments that can be considered relevant moderators of treatment outcomes [36].

In our small dataset there was a trend with a moderate effect size toward higher baseline BMIs in patients who adhered to full treatment, which was consistent with the findings in the ANTOP study, where higher baseline BMIs were found to be associated with better outcomes [37]. Further, depression (represented by the current use of antidepressants) appeared to be an important factor contributing to the possibility of unfavorable treatment outcomes. This is consistent with a systematic review that assessed the influence of psychiatric comorbidities on weight gain in AN finding that depression was the comorbid disorder with the most-obvious negative influence on treatment outcomes [38]. When depression was assessed in terms of depressive symptoms (BDI) or MINI-diagnoses before start of treatment, there were however no differences between patients completing treatment or not. Still, when examining the high baseline BDI scores in our sample it is uncertain whether depression was adequately treated prior to CBT-E treatment.

### Treatment-related reasons

We further questioned if CBT-E was too demanding for this group of patients, due to the high percentage of severe comorbidities and previously failed AN treatment attempts. However, our impression is that dropout is not specific for CBT-E but a problem itself when offering treatment to patients with AN, and we have no indications that other treatments would have resulted in lower dropout rates [39]. All eating-disorder treatments that do not omit important elements such as normalizing eating and monitoring weight are strenuous for the patients, which is inherent in eating disorders rather than in the treatment technique. Flexibility while ensuring fidelity should be considered when implementing evidence-based treatment protocols [40]. Kendall and Frank describe *flexibility within fidelity* as implementation of at treatment protocol “in a manner that contains the core ingredients to attain fidelity, but adapts its implementation to be in sync with individual clients’ presentations.” A vital question would then be what are the key components and the adaptable peripheries of CBT-E. The CBT-E protocol provides relatively good advice regarding the flexible application of techniques and addressing different maintenance factors. At the same time, some aspects of the treatment such as self-monitoring eating and weight and biweekly and weekly sessions are considered essential to ensure fidelity and positive outcomes. The protocol lacks specific techniques for handling trauma-related comorbidities, and has a somewhat restricted focus on emotional maintaining factors. Consequently, the question arises on how to work with maladaptive emotional responses and trauma symptoms in CBT-E in a fidelity-consistent way.

The most-often-reported reason for dropout in this study was early difficulty in adapting to the treatment method. When specified, a gap was observed between which aspects the therapists considered as essential for treatment fidelity (e.g., self-monitoring and weighing) and the patients’ experience of and willingness to apply these methods. Explaining the rationale for the usefulness and necessity of applying different methods to induce changes was focused on by the therapists. However, several patients still reported that the treatment was not adapted sufficiently well to their needs. Some patients also interpreted their reluctance toward these methods as being related to their general ambivalence to challenging their eating disorder, since avoidance becomes more difficult with close monitoring.

About 30% of the included patients had previously undergone CBT-E. This raises the question of whether CBT-E should again be provided or if other treatment approaches should be offered, which might include addressing aspects such as comorbid affective disorders. This question has no obvious answer, but treatment manuals for specific psychological disorders are generally found to be applicable, even in the presence of comorbidities [40]. Within the CBT-E framework, battling an eating disorder demands focus and persistency that might be hampered if comorbidities are addressed too broadly. By now, a sequential approach is often taken, such as addressing PTSD only after the eating disorder is stabilized or treating severe depression—which is a barrier to eating-disorder treatment—before starting CBT-E [15].

We still believe that specific evidence-based approaches are important for treating eating disorders, rather than using an eclectic approach involving several elements of different therapies. This is due to the need of staying focused because of the high levels of ambivalence and anxiety related to changes in diet and weight in eating disorders and the drive for avoidance. However, for some of the most treatment resistant patients with failed prior attempts of CBT-E and reluctance to try again, it could be meaningful to offer another treatment approach, such as SSCM [41].

Beyond the obvious limitation of the small sample, other factors might have affected the study outcome. The patient self-reporting of traumatic experiences prior to treatment and establishing a therapeutic relationship might not be comprehensive. PTSD diagnoses do not capture subthreshold symptoms for trauma-related behavior that might have been factors contributing to the complicated nature of the treatment and increased the risk of premature treatment cessation. Further, we did not record treatment sessions to assess adherence to the method, and it is therefore possible that the clinicians in the present study spent less time than necessary in addressing ambivalence before administering CBT-E, and also during therapy. On the other hand, ambivalence was discussed regularly in the weekly team meetings. The lack of follow-up data beyond the end of treatment was also a limitation.

## Conclusions

Our previous implementation study [13] was consistent with other evidence, and indicated that CBT-E is a suitable treatment method for large groups of adult outpatients with AN. However, maintaining favorable results in everyday clinical treatment and maintaining an evidence-based treatment setting mostly based on CBT-E at a tertiary treatment center has been challenging. Probably our experiences when implementing CBT-E are not unique. The high dropout rate in the present study might contribute to the discussion on how to treat complex patients with AN. Our findings suggest that different patient subgroups require individualized treatment plans beyond the broad version of CBT-E. Most of the included patients had already undergone unsuccessful treatment at a secondary health care unit. Beyond the important clinical question on how to address the complex comorbidities in AN, there are currently no guidelines on how to treat patients who have dropped out from previous CBT-E treatment or have not responded to CBT-E. To help a broader group of patients with AN, an integrated treatment approach based on an expanded CBT-E model should be developed, which includes advice for handling comorbidities.

Considering the high dropout rate in our study and the large number of patients who did not even start treatment, the strategies for engaging patients in therapy need to be improved. This study indicated that patients who adhered to the full CBT-E treatment course had a favorable outcome. Implementing evidence-based treatments in clinical settings outside research facilities is indisputably necessary, but this remains challenging. We suggest that there is a need for clinical research performed in routine clinical settings to reveal the factors underlying failures in engaging patients in treatment and those that can prevent premature dropout. Treating complex AN requires focus and dedication from both the therapist and the patient. However, it also important to note that commitment is also required at a systemic level.

## Data Availability

Data are available from the corresponding author upon reasonable written request.

## Abbreviations

AN: Anorexia nervosa
ANTOP: Anorexia Nervosa Treatment of OutPatients
BAI: Beck anxiety inventory
BDI: Beck depression inventory
BMI: Body mass index
CBT: Cognitive behavioral therapy
CBT-E: Enhanced cognitive behavioral therapy
CIA: Clinical impairment assessment questionnaire
DSM-5: Diagnostic and Statistical Manual of Mental Disorders, Fifth Edition
EDE-Q: Eating Disorder Examination Questionnaire
GSI: Global Severity index
MANTRA: Maudsley Model of Anorexia Nervosa Treatment for Adults
MINI: Mini-International Neuropsychiatric Interview
NHS: National Health Service
NICE: National Institute for Health and Care Excellence
PTSD: Posttraumatic stress disorder
SCL-90-R: Symptom checklist-90–revised
SSCM: Specialist supportive clinical management
SWAN: Strong without anorexia nervosa
WAI-SF: Working alliance inventory–short form

## References

[1] Watson HJ, Bulik CM (2013). Update on the treatment of anorexia nervosa: review of clinical trials, practice guidelines and emerging interventions. Psychol Med.

[2] Solmi M, Wade TD, Byrne S, Del Giovane C, Fairburn CG, Ostinelli EG (2021). Comparative efficacy and acceptability of psychological interventions for the treatment of adult outpatients with anorexia nervosa: a systematic review and network meta-analysis. Lancet Psychiatry.

[3] Hay PJ, Claudino AM, Touyz S, Abd Elbaky G. Individual psychological therapy in the outpatient treatment of adults with anorexia nervosa. Cochrane Database Syst Rev. 2015(7):CD003909. 10.1002/14651858.CD003909.pub2 PMC6491116 26212713

[4] Dalle Grave R, El Ghoch M, Sartirana M, Calugi S (2016). Cognitive behavioral therapy for anorexia nervosa: an update. Curr Psychiatry Rep.

[5] Fairburn CG, Cooper Z, Doll HA, O'Connor ME, Palmer RL, Dalle GR (2013). Enhanced cognitive behaviour therapy for adults with anorexia nervosa: a UK-Italy study. Behav Res Ther.

[6] Zipfel S, Wild B, Gross G, Friederich HC, Teufel M, Schellberg D (2014). Focal psychodynamic therapy, cognitive behaviour therapy, and optimised treatment as usual in outpatients with anorexia nervosa (ANTOP study): randomised controlled trial. Lancet.

[7] Byrne S, Wade T, Hay P, Touyz S, Fairburn CG, Treasure J, et al. A randomised controlled trial of three psychological treatments for anorexia nervosa. Psychol Med. 2017:1–11. 10.1017/S0033291717001349 28552083

[8] National Institute for Health and Care Excellence [NICE]. Eating disorders: recognition and treatment. (NC69). 2017. Retrieved from www. nice.org.uk/guidance/ng69. 28654225

[9] Weissman RS, Frank GKW, Klump KL, Thomas JJ, Wade T, Waller G (2017). The current status of cognitive behavioral therapy for eating disorders: marking the 51st annual convention of the association of behavioral and cognitive therapies. Int J Eat Disord.

[10] Waller G (2016). Treatment protocols for eating disorders: clinicians' attitudes, concerns, adherence and difficulties delivering evidence-based psychological interventions. Curr Psychiatry Rep.

[11] Peterson CB, Becker CB, Treasure J, Shafran R, Bryant-Waugh R (2016). The three-legged stool of evidence-based practice in eating disorder treatment: research, clinical, and patient perspectives. BMC Med.

[12] Schaffner AD, Buchanan LP (2008). Integrating evidence-based treatments with individual needs in an outpatient facility for eating disorders. Eat Disord.

[13] Frostad S, Danielsen YS, Rekkedal GA, Jevne C, Dalle Grave R, Ro O (2018). Implementation of enhanced cognitive behaviour therapy (CBT-E) for adults with anorexia nervosa in an outpatient eating-disorder unit at a public hospital. J Eat Disord.

[14] Danielsen YS, Ardal Rekkedal G, Frostad S, Kessler U (2016). Effectiveness of enhanced cognitive behavioral therapy (CBT-E) in the treatment of anorexia nervosa: a prospective multidisciplinary study. BMC Psychiatry.

[15] Fairburn CG (2008). Cognitive behavior therapy and eating disorders.

[16] American Psychiatric Association. Diagnostic and Statistical manual of mental disorders, 5th ed. Washington, DC (2013).

[17] Fairburn CG, Beglin S, Fairburn CG (2008). Eating disorder examination questionnaire. Cognitive behavior therapy and eating disorders.

[18] Bohn K, Doll HA, Cooper Z, O'Connor M, Palmer RL, Fairburn CG (2008). The measurement of impairment due to eating disorder psychopathology. Behav Res Ther.

[19] DeRogatis LR, Unger R. Symptom checklist-90-revised. Corsini Encyclopedia of Psychology. 2010:1–2.

[20] Beck AT, Steer RA, Brown GK (1996). BDI-II: beck depression inventory, manual.

[21] Beck AT, Epstein N, Brown G, Steer RA (1988). An inventory for measuring clinical anxiety: psychometric properties. J Consult Clin Psychol.

[22] Tracey TJ, Kokotovic AM (1989). Factor structure of the working alliance inventory. Psychol Assess J Consult Clin Psychol.

[23] Sheehan DV, Lecrubier Y, Sheehan KH, Amorim P, Janavs J, Weiller E, et al. The Mini-International Neuropsychiatric Interview (M.I.N.I.): the development and validation of a structured diagnostic psychiatric interview for DSM-IV and ICD-10. J Clin Psychiatry. 1998;59 Suppl 20:22–33. 9881538

[24] Cohen J. statistical power analysis for the behavioral sciences. 2 ed. Routledge, New York (19880.

[25] Ro O, Reas DL, Stedal K (2015). Eating disorder examination questionnaire (EDE-Q) in Norwegian adults: discrimination between female controls and eating disorder patients. Eur Eat Disord Rev.

[26] Fassino S, Piero A, Tomba E, Abbate-Daga G (2009). Factors associated with dropout from treatment for eating disorders: a comprehensive literature review. BMC Psychiatry.

[27] Byrne SM, Fursland A, Allen KL, Watson H (2011). The effectiveness of enhanced cognitive behavioural therapy for eating disorders: an open trial. Behav Res Ther.

[28] Mountford VA, Allen KL, Tchanturia K, Eilender C, Schmidt U (2021). Implementing evidence-based individual psychotherapies for adults with eating disorders in a real world clinical setting. Int J Eat Disord.

[29] Linardon J, Hindle A, Brennan L (2018). Dropout from cognitive-behavioral therapy for eating disorders: a meta-analysis of randomized, controlled trials. Int J Eat Disord.

[30] Byrne S, Wade T, Hay P, Touyz S, Fairburn CG, Treasure J (2017). A randomised controlled trial of three psychological treatments for anorexia nervosa. Psychol Med.

[31] Wild B, Friederich HC, Gross G, Teufel M, Herzog W, Giel KE (2009). The ANTOP study: focal psychodynamic psychotherapy, cognitive-behavioural therapy, and treatment-as-usual in outpatients with anorexia nervosa–a randomized controlled trial. Trials.

[32] Trottier K, MacDonald DE (2017). Update on psychological trauma, other severe adverse experiences and eating disorders: state of the research and future research directions. Curr Psychiatry Rep.

[33] Mitchell KS, Scioli ER, Galovski T, Belfer PL, Cooper Z. Posttraumatic stress disorder and eating disorders: maintaining mechanisms and treatment targets. Eat Disord. 2021:1–15. 10.1080/10640266.2020.1869369 33411646

[34] Brewerton TD (2019). An overview of trauma-informed care and practice for eating disorders. J Aggress Maltreat Trauma.

[35] Trottier K, Monson CM, Wonderlich SA, Olmsted MP (2017). Initial findings from project recover: overcoming co-occurring eating disorders and posttraumatic stress disorder through integrated treatment. J Trauma Stress.

[36] Zeeck A, Herpertz-Dahlmann B, Friederich HC, Brockmeyer T, Resmark G, Hagenah U (2018). Psychotherapeutic treatment for anorexia nervosa: a systematic review and network meta-analysis. Front Psychiatry.

[37] Wild B, Friederich HC, Zipfel S, Resmark G, Giel K, Teufel M (2016). Predictors of outcomes in outpatients with anorexia nervosa - Results from the ANTOP study. Psychiatry Res.

[38] Eskild-Jensen M, Stoving RK, Flindt CF, Sjogren M (2020). Comorbid depression as a negative predictor of weight gain during treatment of anorexia nervosa: a systematic scoping review. Eur Eat Disord Rev.

[39] Schmidt U, Magill N, Renwick B, Keyes A, Kenyon M, Dejong H (2015). The Maudsley outpatient study of treatments for anorexia nervosa and related conditions (MOSAIC): comparison of the maudsley model of anorexia nervosa treatment for adults (MANTRA) with specialist supportive clinical management (SSCM) in outpatients with broadly defined anorexia nervosa: a randomized controlled trial. J Consult Clin Psychol.

[40] Kendall PC, Frank HE. Implementing evidence-based treatment protocols: flexibility within fidelity. Clin Psychol (New York). 2018;25(4). 10.1111/cpsp.12271 PMC6329472 30643355

[41] Touyz S, Le Grange D, Lacey H, Hay P, Smith R, Maguire S (2013). Treating severe and enduring anorexia nervosa: a randomized controlled trial. Psychol Med.

